# Mutations in DISC1 alter IP_3_R and voltage-gated Ca^2+^ channel functioning, implications for major mental illness

**DOI:** 10.1042/NS20180122

**Published:** 2021-12-07

**Authors:** Ann R. Rittenhouse, Sonia Ortiz-Miranda, Agata Jurczyk

**Affiliations:** 1 Program in Neuroscience, NeuroNexus Institute, Department of Microbiology and Physiological Systems, University of Massachusetts Medical School, Worcester, MA 01605, U.S.A.; 2 Agata Jurczyk, Black Diamond Therapeutics, One Main Street, 10th Floor, Cambridge, MA 02142, U.S.A.

**Keywords:** AMPKalpha, Ca2+ signaling, CaValpha1 subunits, exocytosis, GSK3beta, SNARE proteins

## Abstract

Disrupted in Schizophrenia 1 (DISC1) participates in a wide variety of developmental processes of central neurons. It also serves critical roles that underlie cognitive functioning in adult central neurons. Here we summarize DISC1’s general properties and discuss its use as a model system for understanding major mental illnesses (MMIs). We then discuss the cellular actions of DISC1 that involve or regulate Ca^2+^ signaling in adult central neurons. In particular, we focus on the tethering role DISC1 plays in transporting RNA particles containing Ca^2+^ channel subunit RNAs, including IP3R1, CACNA1C and CACNA2D1, and in transporting mitochondria into dendritic and axonal processes. We also review DISC1’s role in modulating IP_3_R1 activity within mitochondria-associated ER membrane (MAM). Finally, we discuss DISC1-glycogen synthase kinase 3β (GSK3β) signaling that regulates functional expression of voltage-gated Ca^2+^ channels (VGCCs) at central synapses. In each case, DISC1 regulates the movement of molecules that impact Ca^2+^ signaling in neurons.

## Introduction

Societies across the globe share a consensus of what constitutes basic, productive human social behavior. This shared reality breaks down in individuals with major psychotic disorders, most notably in schizophrenia (Schz). The inability of most schizophrenics to function as independent adults, due to hallucinations and delusions, cognitive disorganization, and in some cases depressed psychomotor functioning (lack of speech, lack of spontaneous movement and various aspects of blunted emotion), profoundly affects the individual’s quality of life and is a burden on their families as well as to society. Despite recognition since early civilization of ‘madness’, the defining of Schz as a distinct psychosis only occurred in the early 20^th^ century [1]. Even with 100 years of study and treatment, the underlying causes of schizophrenia have been difficult to identify. Epigenetic and epistatic factors, as well as expression of unknown genes that protect against mental illness, obscure the influence of genetic inheritance.

However, this dearth of information is changing. A series of landmark genome-wide association studies (GWAS) have provided new insights into the genetic underpinnings of the disorder. Surprisingly, the psychotic disorders, bipolar disorder (BPD), major depression disorder (MDD), and Schz share sufficient overlapping GWAS hits that more recent bioinformatics studies have pooled thousands of individuals under the general heading of major mental illness (MMI). Identification of susceptibility genes common to BPD, MDD, and Schz is consistent with the observation that these three disorders have overlapping symptoms and treatment strategies. Interrogation of GWAS [2–6], as well as chromatin [7] transcriptome [8,9], exome (the protein coding region of the genome) [10], signaling pathway database analyses [4,11], and recent polygenic analyses [10,12–13], have revealed hundreds of genes with single nucleotide polymorphisms (SNPs) that associate with these three major psychotic disorders.

From interrogating tens of thousands of human genomes, bioinformatics studies have found associations between common variants in genes, where each SNP contributes mild susceptibility for Schz, however in combination with multiple other susceptibility loci, appear sufficient to give rise to Schz. Rare variants have higher penetrance in up to 20% of Schz cases. These mutations nearly always give rise to symptoms and usually are idiopathic in that the parents of the affected individual are healthy. Lastly, a small number of families worldwide have been identified that carry a gene disruption resulting in high penetrance for MMI in multiple generations. Interestingly, the heritability incidence for Schz is estimated to be at least 70% [14–17] and as high as 80% between twins [18], yet our understanding of how heritability functionally contributes to the etiology of MMI is at best superficial. One strategy to gain functional insight is to examine the biology of these identified genes in healthy individuals to begin to understand their role in cognition.

**Figure 1:**
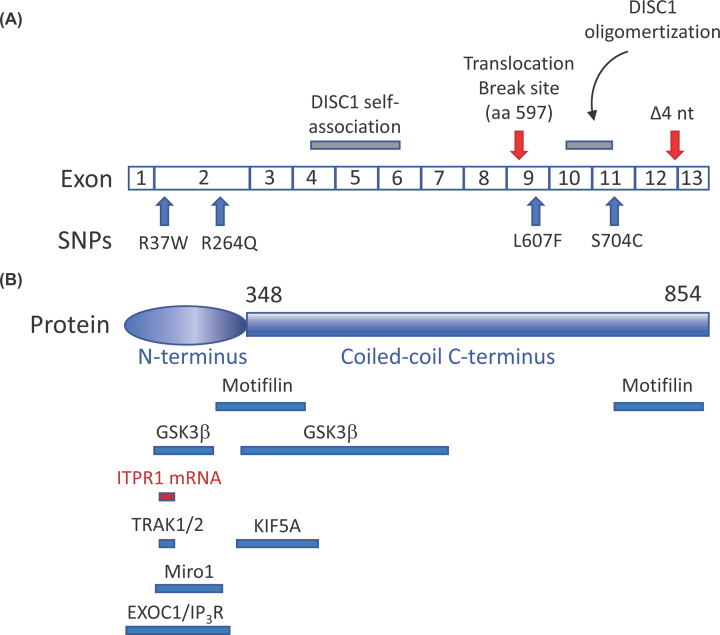
Schematics of DISC1 organization and key interaction sites (**A**) DISC1 mRNA is composed of 13 exons. Red arrows denote the translocation break site in the Scottish family and the 4-nucleotide deletion from the American family. Blue arrows show RNA locations that give rise to amino acid substitutions, which result in altered nerve cell function and/or behavior. Gray bars identify RNA sequences that give rise to DISC1 self-association and oligomerization. (**B**) Schematic of full length 854 amino acid DISC1 protein showing its putative globular or disordered head region and a long coiled-coil region. The relative bindings sites are shown for key proteins (blue bars) and mRNA (red bar).

In this review, we highlight the gene, Disrupted in Schizophrenia 1 (*DISC1*), the earliest gene discovered that associates with MMI disorders. Its discovery marked the beginning of research on the molecular basis of psychiatric disorders. Jacob et al. (1970) identified a large Scottish family in which multiple generations of family members (more than four generations) exhibited high penetrance for MMI [19]. As many as 70% of the carriers exhibit major psychotic symptoms associated with BPD, MDD, and/or Schz [20–26]. These family members carry a balanced translocation from chromosome 1q42.1 to 11q14.3 [23]. The break point, identified in 2000, occurs within the *DISC1* gene resulting in expression of a truncated DISC1 protein that can exhibit intergenic splicing with TRAX or translin-associated factor X (TSNAX) [23]. Many DISC1-binding partners have been identified; how its truncation may alter its interactions with these proteins to precipitate MMI is under intense investigation (Figure 1).

In the following sections, we briefly review the strengths of DISC1 as a useful model system for interrogating the cellular basis of MMI. We then summarize *DISC1* gene organization, how its expression affects neuron morphology, and its role in moving organelles along microtubules. Lastly, we focus on the role DISC1 plays in regulating calcium (Ca^2+^) physiology at adult central synapses by examining its control over IP_3_ receptors, located in mitochondria-associated endoplasmic reticulum (ER) membrane (MAM), and in regulating voltage-gated Ca^2+^ channel (VGCC) subunit expression at synapses. While DISC1 has been implicated in binding many proteins to affect multiple cellular functions throughout ontogeny, this review is not exhaustive, but rather, attempts to pull together studies examining cellular changes in Ca^2+^ signaling in human neurons and human induced pluripotent stem cells (iPSCs) as well as in rodent DISC1 model systems. For earlier insights on DISC1 function and on its regulation of Ca^2+^ signaling through glutamate receptors, we refer the reader to a number of excellent earlier reviews [27–31]. Additionally, while altered Ca^2+^ signaling may create a broad-based susceptibility to certain MMI, we are aware that alternate causes of MMI may arise from disruption in other basic physiological processes including immune functioning. See recent reviews for insightful discussion of immune contributions to MMI [32,33].

## DISC1 is a useful model to study MMI

The translocation first revealed in the *DISC1* gene is a historic example of a rare genetic event occurring in the Scottish family where the proband did not have Schz. Genetic linkage in this pedigree associates with Schz (LOD = 3.6) as well as a broad phenotype (LOD 7.1) for BPD, MDD, and Schz [23,24]. An ever growing body of data, summarized below, point to a role for DISC1 in normal cognition, and as such, serves as a useful model for probing underlying changes that occur in MMI.

### Karyotype analysis

Extensive Karyotype analysis of the Scottish family pedigree revealed that family members carry either no alteration, or a combination of three cytogenetic abnormalities observed by Karyotype analysis. These include: (1) a balanced translocation t(1q42.1 to 11q14.3); (2) a large constriction in the q arm below the centromere of chromosome 1 but above the translocation site; and/or (3) a Robertsonian translocation, characterized by a break at the asymmetric centromere of group D chromosomes (chr13–15), where the long arms fuse to form a single long chromosome [19,23,24]. A large research group at the University of Edinburgh has concentrated on tracking members within the family tree carrying the *t*(1:11) balanced translocation *only* and found that this genotype tracks with susceptibility to MMIs, specifically BPD, MDD, and Schz, through multiple generations; see Porteous et al., 2014 [34]. These findings support the argument that the translocation alone is sufficient for increased susceptibility to MMI.

### Additional families with mutations in *DISC1*

While the possibility that *DISC1* confers susceptibility to MMI generated initial excitement, it was not clear that the psychosis associated with the Scottish family was due to (1) nonfunctional DISC1, (2) other genes disrupted by the translocation, (3) coprecipitation of multiple disrupted genes, or (4) altered chromatin structure. One way to resolve the importance of the DISC1 truncation was to find other families with altered *DISC1* sequence with high penetrance for MMI. Additional families have been identified that only carry a small deletion of nucleotides in the *DISC1* gene that results in premature termination, including Finnish [35–40], American [41,42], Chinese [43,44], and Taiwanese [45] families. These findings suggest that the truncated (*tr*) *DISC1*, observed in the Scottish family, rather than other genes within or near the disrupted regions of chromosomes 1 and 11, is sufficient for conferring MMI susceptibility.

### *DISC1* as a susceptibility gene in genetic studies of MMI

In early GWAS for MMIs, *DISC1* was not identified as a susceptibility gene raising questions about its function and its use as a model system for MMI. Additionally, no other genetic study implicated *DISC1* in MMI due to rare exonic variation, rare copy number variation (CNV), or common variations [10,46–48]. Initially, meta-analysis of common *DISC1* SNPs also found no evidence of genome-wide association with Schz [49]. However, with increased numbers of samples in databases, recent meta-analyses identified *DISC1* polymorphisms and CNV that associate with Schz [50–52]. Moreover, smaller, more focused GWAS have identified *DISC1* as a susceptibility gene for MMI, including six ultrarare non-synonymous amino acid substitutions in DISC1 [53,54], rare missense mutations found in a Swedish cohort [55], and an excess rare variant in exon 11 [56].

### *DISC1* mutations and association with MMI

A limitation of GWAS and many CNV studies of MMI susceptibility genes is that they do not identify whether any of these variants affect function [6]. However, a number of DISC1 variants that cause cellular and behavioral abnormalities in model systems also associate with Schz [53] and recurrent MDD [57]. Most notably, an ultrarare DISC1 variant R37W in a case of MDD transmitted to two affected offspring [53]. Additionally two SNPs in intron 9 (rs821577 and rs2295959) show female-specific associations with anxiety, depression, and neuroticism in elderly Scottish subjects [58], and female-specific association with Schz in Han Chinese subjects [43], and with Schz in a Japanese population [59]. Most useful for examining the cellular role of DISC1 are several different amino acid substitutions of Leu^607^, located in exon 9 and conserved across humans, mice, rats, pufferfish, and zebrafish (Figure 1A). One of these, a Leu^607^Phe substitution correlates with schizoaffective disorder in an American family [42].

### Broad versus specific behavioral phenotypes found with mutant DISC1

Because of the high penetrance of multiple forms of MMI in affected families carrying *trDISC1* and recent GWAS identifying *DISC1* as a general versus specific susceptibility gene for a particular illness, concern exists that its interrogation will yield only superficial understanding of the etiology of Schz. However, DISC1 mutations do correlate with various brain endotypes including behavioral differences in anhedonia and frontal-lobe-associated memory; electrophysiological changes observed in auditory-event-related potentials (ERPs); and anatomical differences in cortical thickness, hippocampal gray matter volume and white matter integrity [60,61]; all changes observed in Schz individuals. Unlike family members without the mutation, 100% of the Scottish family members tested, who carried the *t*(1:11) translocation, exhibit increased latency and/or smaller amplitude P300 responses [24], a quantitative measure of cerebral ERPs associated with attention and memory processes [34]. Different *trDisc1* mouse models exhibit similar changes in behavior, brain circuitry, and synaptic transmission as Schz individuals. If DISC1 confers a more generalized versus selective risk to MMI, studying its function should lead to identification of specific signaling pathways that become disrupted in different psychotic diseases. Indeed, DISC1 interacts with a plethora of Schz susceptibility genes, reinforcing the research strategy of understanding DISC1 function to gain insight into the etiology of MMI.

## Gene organization and expression of DISC1

The *DISC1* gene is 414.3 kb with 13 exons located in chromosome 1; it is the only known member of its family. Of note, *DISC2* is a non-protein coding lncRNA transcribed from the 3′ region of *DISC1* and made up of one large exon located antisense to exon 9 of *DISC1*. Little is known about *DISC2’s* function though it is hypothesized to regulate *DISC1* expression [23]. The break point within the *DISC1* gene of the Scottish family (Figure 1) occurs within intron 8 resulting in the translocation of exons 9–11 to chromosome 11 as well as the majority of *DISC2*. Full-length DISC1 is 854 amino acids long with an amino-terminus head domain (amino acids 1–347) that contains nuclear localization signals (Figure 1B). Its C-terminus domain (amino acids 348–854), encoded by exons 3-13, contains coiled coil sequences [30]. DISC1 has binding sites for a large number of proteins, including PDE4B, PCM1, NDE1, IP3R1, and glycogen synthase kinase 3β (GSK3β), which are independently implicated as genetic risk factors for Schz and related MMI [27]. Key SNPs have been found either proximal to regions encoding DISC1 protein interaction domains or are predicted exonic splicing enhancer sites, identifying functionally relevant regions of interest for interrogation [62–64]. At least 50 different transcripts of *DISC1* are expressed dynamically over time in various brain regions [44,65,66]. Different cell types in adult cortical brain regions express unique profiles of *DISC1* transcripts, which in turn yield, or are predicted to yield, unique DISC1 interactomes for a given cell type over a lifetime with greatest interest in hippocampal and cortical glutamatergic pyramidal neurons and GABA inhibitory interneurons [53,62,65,67,68].

The large number of transcripts may help explain how DISC1 serves so many neuronal functions. Different molecular weight proteins stain positive for DISC1 in Western blot analysis [see [30]], suggesting that multiple transcripts of *DISC1* express as protein. DISC1 is enriched in the adult prefrontal cortex, hippocampus and striatum, brain areas identified as important in MMI [69]. Within these areas, DISC1 plays major roles in neuronal proliferation [70], cell migration, nucleokinesis [71–73], axonal transport, neurite outgrowth, dendrite arborization, spine morphology, synaptogenesis, synaptic transmission, and synapse maintenance [70], [see [27]]; all processes that are disrupted in Schz. Moreover, proteomics and interactome studies have identified proteins that interact with DISC1 protein [70,71]. Despite some concerns about specificity of DISC1 antibodies there seems to be consensus on where DISC1 is expressed and which proteins interact with DISC1. As more reagents and model systems are utilized in future studies, further comparisons among studies on DISC1 expression and function will occur. Moreover, commercial DISC1 antibodies are now available allowing multiple labs to use the same reagents, facilitating comparison of results gathered from probing DISC1’s functional interactions in different model systems.

Postmortem analysis of human hippocampus and dorsolateral prefrontal cortex revealed that expression of short *DISC1* variants is higher *in utero* than postnatally [66]. Though its levels decrease postpartum, DISC1 expression remains critical in adult brain for proper cytoskeletal function conveying neuronal polarity [74,75], axonal transport [63,75–77] and synaptic function [78–81]. These processes are notable since they participate in both development as well as adult synaptic plasticity. Enrichment of short protein isoforms of DISC1 occurs in Schz brains [66], suggesting that persistence of their elevated levels may disrupt both brain development as well as adult cognition. More research is needed to understand the significance of the widespread distribution of DISC1 splice variants [11,65] [see also [72]] as well as, which isoforms dynamically interact with, and affect the function of various proteins in different subcellular locations over a lifetime [82–84] (Figure 1B). With so many binding sites for proteins with different cellular roles, one can begin to imagine how mutant or *tr*DISC1 could create unique pathologies in different cell types [85].

A notable feature of DISC1 is that in addition to binding many proteins, it is its own binding partner [66]. Co-immunoprecipitation studies show that DISC1 self-associates through a domain proximal to where the *t*(1,11) break occurs in the Scottish family [86]. *tr*DISC1 functions as a dominant negative, binding to full-length DISC1 to form insoluble aggregates (Figure 1A). These aggregates result in lowered DISC1 expression levels in human iPSCs and in mouse hippocampus, as well as abnormal cognitive function [76,87]. DISC1 aggregates are recruited to aggresomes that also attract soluble DISC1, but not to Golgi, ER or endocytic pathways [76,88,89]. Aggregated DISC1 shows minimal ability to return to the cytosolic fraction. Rather, it is degraded by the autophagy pathway. Insoluble DISC1 has been found in brain tissue of patients suffering from MMI [76,88]. These aggresomes are reminiscent of protein precipitate that accompany cognitive decline in neurodegenerative diseases, such as in Parkinson’s Disease, Alzheimer’s Disease, and frontotemporal dementia [90].

## Known functions of DISC1

How truncation of DISC1 contributes broad risk for MMI is a key question that has been probed intensively for more than 20 years. Key morphological changes in CNS neurons provide clues as to how loss of functional DISC1 may increase susceptibility to MMI. Decreased numbers of synaptic spines in cortical regions of autopsied Schz brains are accompanied by elongated dendrites with reduced branching [91]. A strikingly similar morphological profile recapitulates in primary cortical neurons from a mouse model that expresses a C-terminally truncated form of *Disc1* under the control of a Tet-off system in cortex, striatum and hippocampus. Expression of *tr*DISC1 results in decreased neurite outgrowth in primary cortical neurons and in reduced levels of SNAP-25 in the forebrain area of young mouse pups [92], suggesting decreases in synapse maturation accompany abnormal arborization. These mice also exhibit sex-dependent changes in behavior, including increased locomotor activity and abnormal social behavior in males while females exhibit impaired spatial reference memory, suggesting that these observed cognitive changes reflect the synaptic changes brought about by *tr*DISC1 expression [92]. Additional interrogation of DISC1’s role in cell morphology reveals that loss of its N-terminus primarily disrupts normal nerve cell proliferation and movement [93], while the C-terminus primarily regulates dendrite morphology and synaptic function. These findings fit with observations that *DISC1* transcripts, located in adult neuronal processes, are primarily C-terminal containing variants, suggesting that active regulation of dendrite morphology by DISC1 contributes to proper synaptic transmission [65,94–97].

**Figure 2:**
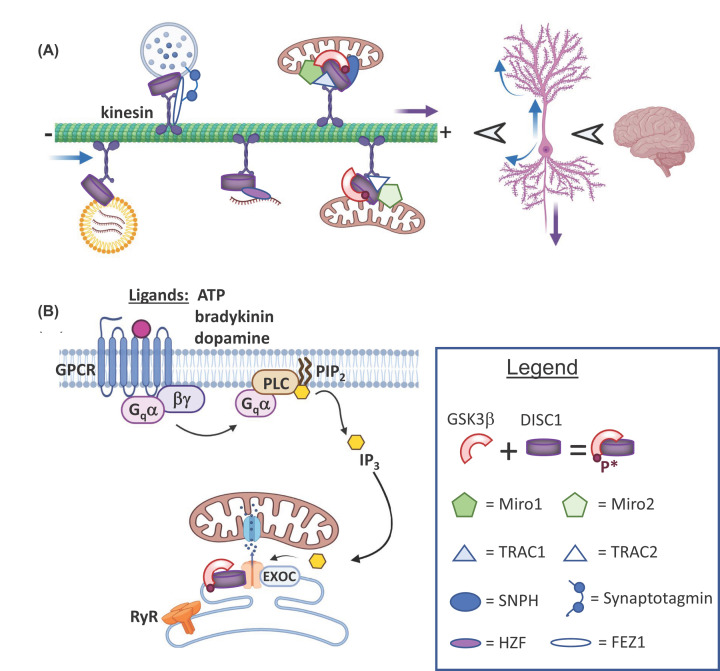
In adult central neurons, DISC1 regulates cargo transport along microtubules (striped green) and Ca^2+^ transfer from ER to mitochondria at MAM (**A**) DISC1 serves as a tether for mitochondria, RNA (brown strands), RNA particles (yellow) and SVs filled with transmitter (pale blue). DISC1 binds to a kinesin motor complex to regulate transport of various cargo into dendrites (blue arrows) and/or nerve terminals (purple arrow). (**B**) DISC1 binding to IP_3_Rs within MAMs lowers the amount of Ca^2+^ transferred via VDAC channels into mitochondria, protecting them from excitotoxicity. DISC1 does not appear to regulate ER Ca^2+^ release by the ryanodine receptor 2 (RYR2).

At the subcellular level, DISC1 is considered a developmental hub protein, because it has no enzymatic function of its own, but rather incorporates into scaffolding where it binds multiple proteins to form signaling complexes that influence many stages of neuronal ontogeny both temporally and spatially [98] [for review; [86,99,100]]. In adulthood, DISC1 serves another critical function as an adaptor protein by tethering cargo to a molecular motor: either kinesin-1 motor complex [101,102] or dynein for anterograde and retrograde movement along microtubules, respectively [96,74,98]. Rather than affecting the rate of movement, DISC1 controls what and when cargo moves along microtubules (Figure 2A). Identified cargos include RNA particles, membraneless granules that contain mRNAs and RNA-binding proteins for controlling localized translation [see [103] for review], as well as mitochondria and synaptic vesicles (SVs) [79,81]. At presynaptic nerve endings, DISC1 regulates expression levels of proteins involved in initiating synaptic transmission [78]. Postsynaptically, DISC1 plays a necessary role in synaptic plasticity by regulating protein movement in and out of the postsynaptic density in a similar manner to regulating cargo movement along microtubules [93,104,105]. Thus, DISC1’s role in spines may be better described as a tethering rather than a scaffolding function where it controls protein movement critical for postsynaptic signaling. At synapses, DISC1’s tethering functions also appear critical in controlling Ca^2+^ signaling. In the following sections, we discuss how DISC1 may alter Ca^2+^ signaling due to its interactions with (1) RNA particles containing Ca^2+^ channel transcripts, (2) IP_3_Rs located in mitochondria-associated ER membranes (MAMs), and (3) VGCCs in nerve terminals.

## DISC1 tethers to molecular motors-specific mRNA particles enriched in transcripts involved in Ca^2+^ signaling and membrane excitability

Proteomic screens for DISC1 interactors identified several RNA-binding proteins, including hematopoietic zinc finger protein (HZF), found in RNA-transport particles [106]. DISC1 has an arginine-rich motif (ARM) region containing a nuclear localization signal (NLS), which may be required for mRNA export from the nucleus. The mRNA of inositol-1,4,5-triphosphate receptor type 1 (*Itpr1*) itself binds DISC1 at an ARM in its N-terminal region. DISC1 binding to *Itpr1* is facilitated by HZF binding to both DISC1 and to a distinct binding site on *Itpr1* mRNA. DISC1 interaction with HZF and kinesin-1 is required for transport of *Itpr1* mRNA along microtubules [101,107]. These findings fit a model proposed by Tsuboi et al. (2015) [106] where kinesin-1 transports the DISC1–HZF–*Itpr1* mRNA complex into distal dendrites of hippocampal neurons similarly to particles formed of mRNAs, RNA-binding proteins, adaptor proteins (such as DISC1) and a molecular motor such as Kinesin-1 [101,107] (Figure 2A).

Evidence in favor of this model is that expression of a dominant-negative mutant of kinesin heavy chain protein KIF5A inhibits transport of *Itpr1* mRNA. Similarly, depletion of DISC1 negatively impacts transport of *Itpr1* mRNA into dendrites, whereas overexpression of DISC1 enhances its transport [106]. These findings are consistent with DISC1 functioning as a cargo adapter, or tether, linking the bound *Itpr1* mRNA to the kinesin-1 complex for transport along microtubules. Whether DISC1 binds to RNA particles containing *Itpr1* mRNA, and/or directly to *Itpr1* mRNA to transit into neuronal processes requires further investigation. Nevertheless, these interactions have functional significance since *Itpr1* mRNA and DISC1 colocalize in hippocampal dendrites where release of Ca^2+^ from IP_3_Rs is critical for initiating long-term changes in synaptic plasticity [108–113]. *Disc1* knockout mice show normal brain cytoarchitecture [68] but exhibit abnormal synaptic activity, impaired maintenance of LTP, and altered emotional behaviors [114]; all functions that involve synaptic plasticity and cognitive behaviors often disrupted in Schz. This phenotype was also observed in hippocampal slices exposed to a cell permeable peptide, which blocks DISC1 binding to *Itpr1* mRNA [106], thus linking DISC1 to Ca^2+^-mediated synaptic plasticity.

In addition to *Itpr1* mRNA, at least several hundred mRNAs, packaged into RNA granules, are transported into dendritic spines [115]. From mouse brain extracts and hippocampal lysates, a subset of these transcripts immunoprecipitate with full-length DISC1 protein when using a C-terminal antibody. These transcripts were at least two-fold enriched in the DISC1 immunoprecipitate compared with an IgG immunoprecipitate. Those identified included the pore-forming subunit of the VGCC Ca_V_1.2 (*Cacna1c*), and its accessory subunit α_2_δ_1_ (*Cacna2d1*); K_V_3.1, a delayed rectifier K^+^ channel (*Kcnc1*), expressed in fast spiking GABA interneurons; and K_V_3.4, a second K^+^ channel family member (*Kcnc4*). The interactions appear direct since *in vitro* RNA binding assays confirmed GST–h*DISC1*–N1 interaction with biotin-labeled 3′UTR mRNA of each gene but not with GST alone or to 3′UTR of other transcripts such as the sodium channel Na_V_2.1 (*Scn2a*), or to the coding sequence of *Cacna2d1* [106]. Taken together, these findings indicate that DISC1 binds to a subset of mRNAs encoding proteins that regulate membrane excitability and Ca^2+^ influx. Decreased functional DISC1 would be predicted to lower transport of mRNA to critical sites, consequently decreasing expression and compromising the normal complement of proteins regulating Ca^2+^-dependent events.

## DISC1 tethers mitochondria to molecular motors to facilitate movement of mitochondria to areas of high metabolic demand

DISC1 also tethers mitochondria to molecular motors (Figure 2A), positively affecting their anterograde axonal transport [116–118]. Knockdown of DISC1 in neurons significantly lowers the number of mitochondria moving along microtubules from 36 to 16% [116,117]. Two non-synonymous C-terminus SNPS of DISC1, Leu^607^Phe and Ser^704^Cys, correlate with MMI and associate with alterations in brain maturation and synaptic function [72,80,119,120]. Leu^607^ lies at the end of DISC1’s putative leucine zipper domain within a region identified as necessary for binding of important factors (e.g., NUDEL, MIPT3, ATF4, ATF5) for neurodevelopment [101]. Leu^607^ also plays a critical role in adult DISC1 function. Expressing the common variants (Leu^607^/Ser^704^) or the Cys^704^ variant in DISC1^−/−^ neurons rescues transport. In contrast, the Leu^607^Phe mutation, which correlates with schizoaffective disorder in an American family [42], is unable to correctly rescue mitochondrial movement. Interestingly, the rare human N-terminal SNP, R37W, also lowers mitochondrial trafficking [121], suggesting that specific residues in both the N- and C-termini of DISC1 may play significant roles in controlling mitochondria movement. Indeed, overexpression of DISC1 raises the numbers of motile mitochondria to 42% compared with 27% [116,117]. However, none of the DISC1 variants alter the velocity of movement, suggesting DISC1 increases the percent of mitochondria in transport rather than controlling the rate of transport, as mentioned above.

Movement of mitochondria by its molecular motor involves dynamic interactions among the kinesin-1 family motor KIF, the trafficking kinesin protein TRAK1, and the transmembrane mitochondrial Rho GTPase adaptor Miro1, which has two GTPase domains each flanked by a Ca^2+^-binding EF hand (Figure 2A). By interacting with proteins within this motor complex, DISC1 promotes Ca^2+^-sensitive anterograde movement of mitochondria [116,122]. A similar motor complex is found in dendrites that relies on DISC1 interaction with Miro2 and TRAK2 for mitochondrial movement [116]. Syntaphilin (SNPH), an additional interaction partner within the modulatory complex, binds directly to microtubules and mitochondria immobilizing them upon exposure to rises in intracellular Ca^2+^[123]. For anterograde movement to occur, DISC1 appears to bind both Miro1 and SNPH keeping them from interacting with one another [123]. With the rise in intracellular Ca^2+^, DISC1 dissociates from the complex allowing SNPH and Miro1 to interact with one another to anchor mitochondria in place (Figure 2A). Furthermore, upon sensing increased Ca^2+^ levels, whether in terminals or dendrites, Miro uncouples the KIF motor from the complex, halting movement of mitochondria along microtubules [123]. Interestingly, in SNPH knockdown studies in cultured cortical neurons, not only does mitochondrial movement increase, but these cells exhibit decreased axonal branching as well as impaired Ca^2+^ buffering in nerve terminals, [124]. These findings suggest that the complement of proteins within a molecular motor complex determines what and when cargo moves or stops, as well as the direction of movement. Dependency on Ca^2+^ to dock mitochondria in place ensures an energy source at regions with metabolic demand. Whether additional proteins act in concert with DISC1 to facilitate loading of mitochondria on to microtubules awaits further interrogation. Taken together, these observations link DISC1’s role in determining morphological characteristics of a neuron’s branching pattern to its Ca^2+^-sensitive role in tethering and facilitating mitochondrial movement to areas of high metabolic demand such as axon arborization and growth cone formation during development and in synapses during neurotransmission.

## DISC1 regulates transfer of Ca^2+^ from ER to mitochondria via MAM

Amino acids 1–350 localize DISC1 to a specialized membrane patch, called MAM, where membrane from the two organelles contact one another (Figure 2B). MAMs are dynamically enriched for stress-related proteins, lipid metabolism enzymes, autophagosome markers, and ion pores including IP_3_Rs and voltage-dependent anion channel 1 (VDAC1), a member of an anion channel family located in the mitochondrial outer membrane that passes both ATP and Ca^2+^ [125]. In cortical neurons, DISC1 colocalizes with IP_3_R1s to MAMs; their depletion by shRNA-*Itpr1* results in less endogenous and flag-tagged recombinant DISC1 in the crude MAM fraction without causing significant decreases in overall DISC1 expression. Surprisingly, DISC1 appears to play no role in the actual tethering of ER to mitochondria at MAMs. Rather, DISC1 blunts IP_3_R1-mediated Ca^2+^ release from ER by binding selectively to the IP_3_ binding domain and modulatory domains 1, 2, & 3 but not to a suppressor domain or transmembrane domains of IP_3_R1s [123,126]. DISC1 has no influence on the intrinsic capacity for Ca^2+^ uptake by mitochondria or on basal ER Ca^2+^ levels [123,126]. Consistent with a role in blunting ER Ca^2+^ release, knockdown of DISC1 using shRNA-*Disc1* inappropriately increases IP_3_-dependent Ca^2+^ transfer through the MAM to mitochondria. Moreover, cortical neurons cultured from DISC1L1 embryos, a mouse line that harbors an impaired *Disc1* locus [127], exhibit significant increases in ER–mitochondrial Ca^2+^ transfer following exposure to IP_3_, which reverses with hDISC1 overexpression. However, the reverse is not the case; mitochondrial Ca^2+^ signals contribute little to altered ER Ca^2+^ dynamics occurring with DISC1 knockdown [123].

Within the MAM, DISC1 also associates with EXOC1, normally a member of the exocyst complex, which targets vesicles to specific docking sites in the plasma membrane [128]. EXOC1 overexpression increases, while its knockdown decreases, ER DISC1 levels in both hippocampal neurons and when recombinant DISC1 is expressed in HEK293 cells [129]. In contrast, ryanodine receptor-mediated Ca^2+^ release, stimulated by caffeine, remains normal when either DISC1 or EXOC1 levels is decreased, supporting a model where DISC1 and EXOC1 regulate ER Ca^2+^ dynamics selectively via IP_3_R1s [129]. In support of this model, the exocyst complex is known to interact directly with IP_3_R3s in neurons to regulate intracellular Ca^2+^ signaling [130]. DISC1 and EXOC1 do not exhibit additive or synergistic effects, but rather function in the same pathway, with DISC1 acting downstream of EXOC1 [129]. This relationship has functional significance since knockdown of either DISC1 or EXOC1 *increases* Ca^2+^ release via IP_3_Rs that normally occurs following ATP-induced purinergic G_q_PCR signaling while overexpression of both decreases the Ca^2+^ signal in HEK293 cells (Figure 2B).

Similarly in neurons, DISC1 modulates Ca^2+^ efflux through IP_3_R1s following G_q_PCR stimulation. DISC1 modulates IP_3_R1-mediated Ca^2+^ transfer from ER to mitochondria following ATP stimulation of hippocampal neurons or bradykinin stimulation of differentiated neuroblastoma CAD cells as well as dopamine stimulation of hippocampal D1/D2 heterodimer receptors [129]. In each case, DISC1 deficiency (shRNA-*Disc1*) results in mitochondrial Ca^2+^ overload following G_q_PCR stimulation. The variety of neurotransmitters that modulate Ca^2+^ transfer from ER to mitochondria indicate a broad role for DISC1 in regulating intracellular Ca^2+^signaling. These findings predict potential pathological Ca^2+^ overload in mitochondria when DISC1 is unable to modulate IP_3_R1 activity. Interestingly, the antipsychotic drug haloperidol reverses this profile of excessive Ca^2+^ transfer from ER to mitochondria when DISC1 levels are low in hippocampal neurons [126]. This finding suggests one of the downstream roles played by haloperidol in stabilizing Schz patients is to promote cellular Ca^2+^ homeostasis.

Lastly in cortical neurons, DISC1 knockdown exaggerates IP_3_-dependent mitochondrial Ca^2+^ overload in response to oxidative stress by H_2_O_2_ or to high levels of corticosterone, leading to excessive ROS production [126]. With Ca^2+^ overload, the mitochondrial membrane potential collapses and its metabolic activity uncouples from its electrical gradient. Park et al. (2017) hypothesize that MAM-localized DISC1 modulates the interpretation of stress into intracellular oxidative stress responses by gate-keeping ER–mitochondria Ca^2+^ crosstalk at the MAM [126]. In response to physiological stress, changes in intracellular [Ca^2+^], oxidative stress by H_2_O_2_, and ROS production may reflect the molecular basis of sensitivity to environmental insults that associate with vulnerability to MMI [126,131]. In summary, the data suggest that DISC1 plays a central role in regulating metabolic status by stimulating mitochondrial travel into distal dendrites and nerve terminals. There, DISC1 localizes to MAMs, where it is part of a macromolecular complex that regulates Ca^2+^ transfer through IP_3_Rs in the ER to mitochondria [129]. The data suggest a link between disrupted DISC1 regulation of Ca^2+^ signaling, whether driven by G_q_PCR-mediated excitotoxicity or oxidative stress, and disrupted cellular metabolic processes that might underlie certain forms of MMI.

## DISC1 indirectly regulates VGCC stability by controlling its phosphorylation by GSK3β and subsequent proteosomal degradation

**Figure 3:**
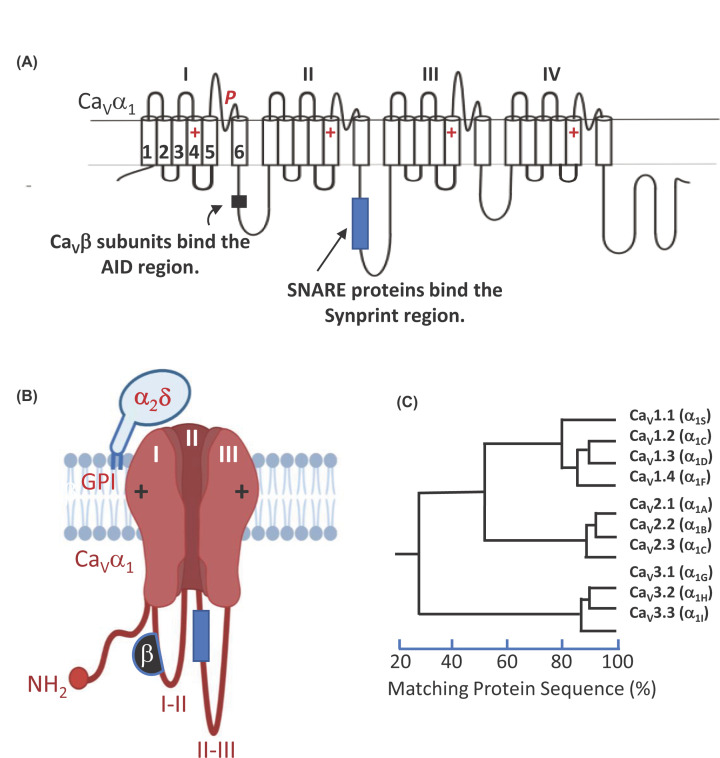
VGCCs are composed of a pore-forming subunit and associated accessory subunits (**A**) Linear topology schematic of pore forming Ca_V_α_1_ subunits. Domains I–IV contain six homologous transmembrane segments where the S1–S4 segments form a voltage-sensor paddle with multiple positively charged amino acids (+) found in S4. S5–56 form the pore with the pore loop (*P*) folding back into the membrane to form the selectivity filter. (**B**) Schematic of a VGCC complex with a Ca_V_α_1_ bound to its Ca_V_β and Ca_V_α_2_-δ subunits (GPI, glycosylphosphatidylinositol anchor). Domain IV and the C-terminal tail have been removed. (**C**) Evolutionary tree of Ca_V_α_1_subunits.

GWAS data reveal that susceptibility genes often fall within the same signaling pathway and/or interact with one another (interactome). As with IP_3_Rs, GWAS and exome studies searching for MMI susceptibility genes identified multiple hits for VGCC subunit genes (Figures 3A-C), including *CACNA1C*, the pore forming subunit of the L-VGCC Ca_V_1.2 and *CACNA1D*, the pore forming subunit of a second L-VGCC, Ca_V_1.3. Also important accessory channel subunits were identified including *CACNB2, CACNA2D1* [5,6,132–134], *CACNG4*, *CACNG5, CACNG6*, and *CACNG8* [135–141]. Moreover, a recent, in depth RNAseq analysis of the *Der1* mouse model, which expresses reduced levels of Disc1, revealed prominent dysregulation of several Ca_V_α_1_ subunits and Ca_V_β2. Subsequent proteomics and pathway analysis identified Ca^2+^ signaling as at risk in Der1 and overlapping with genetic risk factors found in Schz GWAS [68].

Particular excitement has surrounded hits on *CACNA1C* since Ca_V_1.2 channels mediate certain forms of excitation–transcription coupling in hippocampus that are integral to long-term changes in central synaptic transmission [142,143]. As mentioned above, DISC1 transports VGCC subunit mRNAs, including *CACNA1C*, into processes [106], linking control of VGCC subcellular location to DISC1. Changes in L-VGCC expression, and/or function as well as location, could precipitate a broad vulnerability to MMI [144]. While L-VGCC mRNA movement into processes decreases with mutant DISC1, Park et al. (2016), using Ca^2+^ imaging methods, found that cortical neurons, deficient in DISC1 or SNPH, exhibited no change in KCl-stimulated Ca^2+^ influx in cell bodies compared with control neurons [123]. The authors argue that L-VGCCs often concentrate in somal regions of neurons and concluded that channel function is unaffected by DISC1. However, their imaging assay may not be sensitive enough to detect a change in Ca^2+^ influx through a particular channel type since cell bodies of central neurons express multiple VGCC types (see Figure 3C), with each type contributing a minority of the Ca^2+^ current. Additionally, all of them inactivate under conditions of sustained KCl-mediated depolarization [145] making it difficult to ascertain whether DISC1 regulates particular VGCCs.

Under normal physiological conditions, Ca^2+^ influx through VGCCs contributes to global Ca^2+^ signaling by positively modulating intracellular Ca^2+^ release and also to local Ca^2+^ microdomains [146] to initiate transmitter release from presynaptic boutons and to mediate postsynaptic integration in dendrites and spines [147]. In contrast with DISC1 binding to and modulating IP_3_R1 activity, currently there is no evidence that DISC1 interacts directly with VGCC subunits to alter channel activity. Rather, DISC1 appears to control channel levels by indirectly regulating phosphorylation of VGCCs by GSK3β [70,78,148]. GSK3β phosphorylates channels in the Ca_V_2 but not in the Ca_V_1 family in cultured hippocampal neurons [148]. However, in colonic smooth muscle GSK3β associates with and phosphorylates Ca_V_1.2b, a common L-VGCC variant with a short C-terminus. Channel phosphorylation by GSK3β appears to target Ca_V_1.2b for ubiquitination followed by proteasomal degradation [149]. Thus, the potential exists for GSK3β-mediated regulation of Ca_V_1.2 levels to occur in neurons. Rather than searching for a DISC1–GSK3β–Ca_V_1.2 connection in presynaptic terminals or cell bodies, probing for DISC1 regulation of Ca_V_1.2 in postsynaptic spines and dendrites may be more relevant. Whereas Ca_V_2 family members mediate transmitter release at most central synapses, Ca_V_1.2 is highly expressed postsynaptically in dendrites of central neurons where it plays roles in synaptic plasticity and excitation–transcription coupling [150].

## DISC1 regulates Ca^2+^-dependent SV fusion and transmitter release by controlling GSK3β activity

**Figure 4:**
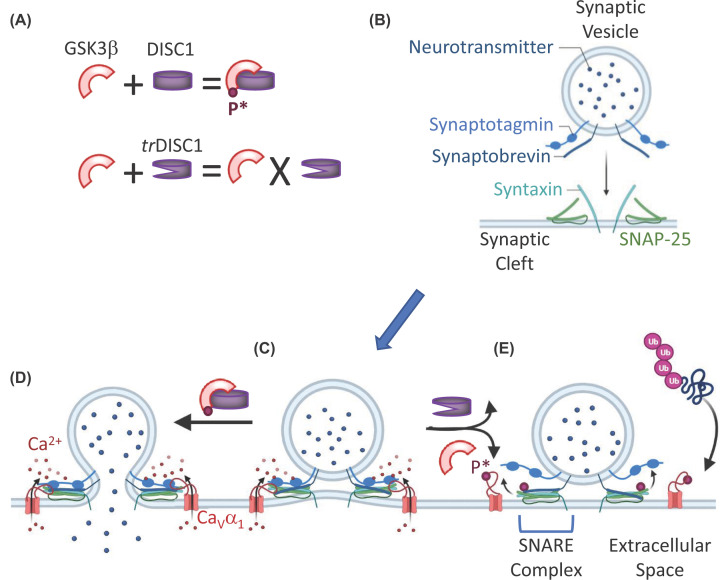
Loss of DISC1 results in uncoupling of VGCCs from neuronal secretion machinery (**A**) DISC1 can bind and phosphorylate GSK3β to tonically inhibit its activity, whereas truncated (*tr*) DISC1 is unable to phosphorylate GSK3β, releasing it from inhibition. (**B,C**) SV proteins interact with terminal membrane proteins to form SNARE complexes (see E) that dock vesicles to release sites. Upon Ca^2+^ influx through VGCCs (**C**), docked vesicles fuse with the plasma membrane to release neurotransmitter (**D**). (**E**) *tr*DISC1 releases GSK3β from inhibition allowing it to phosphorylate (maroon spheres) VGCCs leading to their dissociation from the secretion machinery. Once uncoupled, VGCCs are ubiquitinated and degraded by the proteosome.

GSK3β is a negatively regulated kinase where tonic phosphorylation of Ser^9^ suppresses its activity [151]. Dephosphorylation of Ser^9^ relieves GSK3β from tonic inhibition allowing it to autophosphorylate Tyr^216^, which activates its enzymatic activity. Activated GSK3β in turn acts on a wide variety of proteins in addition to L-VGCCs, including proteins involved in SV fusion and transmitter release (Figure 4). Mao et al (2009) investigated what proteins are downstream from DISC1 that disrupt transmitter release and synaptic plasticity in adult hippocampus and found DISC1’s N-terminus (residues 211–225) binds GSK3β directly to impede phosphorylation of Tyr^216^ [70]. GSK3β also binds DISC1 in its α-helical coiled-coil-terminal region (residues 356–595). Point mutations in either of these two regions disrupt DISC1 binding to GSK3β. Notable examples include N-terminus DISC1 mutants Q31L and L100P in mice and R264Q in humans (Figure 1B) exhibit decreased binding to GSK3β [152,153]. A C-terminal D453G mutation in mice does not alter DISC1 expression levels but does disrupt its binding to GSK3β, decreasing it more than 50% in whole brain homogenates [154]. The consequences of sustained GSK3β activation in nerve processes include suppression of glutamate release, loss of LTP in hippocampal neurons, and altered behaviors associated with cognitive function [70,148,155].

One obvious place to look for downstream targets mediating DISC1–GSK3β regulation of adult mouse behavior is at central synapses. Zhu et al. (2010) examined whether, and if so, how GSK3β might affect excitation–secretion coupling using the fluorescent protein FM4-64. FM4-64 is taken up by SV where it fluoresces in this acidic environment but is quenched upon release into the neutral extracellular space [148]. They found that recombinant EGFP-tagged (wt)GSK3β inhibits presynaptic vesicle exocytosis, measured from dissociated hippocampal neurons during Hi K^+^ stimulation. When GSK3β is activated indirectly by wortmannin, wtGSK3β neurons also exhibited decreased whole-cell P/Q (Ca_V_2.1) current compared with neurons transfected with dnGSK3β. Concomitantly, the magnitude of intracellular Ca^2+^ signals, measured with Fluo3AM, coincided with the changes in P/Q current amplitude. VGCCs are composed of four repeating domains connected by intracellular loops (Figure 3A). Previously protein kinase C (PKC) and calmodulin-dependent protein kinase II (CaMKII) were shown to phosphorylate serine residues within a region of the II–III linker, called the synprint region of N-(Ca_V_2.2) as well as P/Q channels [156]-[157]. The synprint region interacts with SV fusion machinery [for review see [158,159]]. Thus, GSK3β may not only decrease Ca_V_2.1 current, but also disrupt excitation–secretion coupling through phosphorylation of the II–III linker (Figures3A-B).

Zhu et al. (2010) probed this possibility by performing immunoprecipitation studies with synaptosomes to test whether GSK3β disrupts synaptobrevin association with syntaxin and SNAP-25 [148]. These three proteins form the SNARE complex, that mediates vesicle fusion with the terminal membrane (Figures 4A-E). Stimulation of GSK3β by wortmannin decreased while the GSK3β inhibitor SB216763 enhanced their co-immunoprecipitation. Moreover, phosphorylation of tyrosine residues within the P/Q channel II–III linker by recombinant GSK3β inhibited channel association with the three SNARE proteins. Using FRET analysis, the authors showed that GSK3β activation also decreased synaptobrevin dissociation from the SV protein synaptophysin l. Each of these interactions is required for a SNARE complex to mediate efficient exocytosis. Unfortunately, the authors did not test whether GSK3β also might phosphorylate SNARE proteins directly. Nevertheless, the data strongly support a model where phosphorylation of the channel’s II–III linker by GSK3β disrupts direct interactions of SNARE proteins with P/Q-VGCCs as well as their complex interactions among themselves; the result of which is decreased activity-dependent exocytosis.

The importance of GSK3β activity in regulating P/Q-VGCC expression is not unique to hippocampal neurons; it also regulates their expression in NPY-expressing arcuate nucleus (ARC-NPY) neurons of the hypothalamus [160]. Western blot findings demonstrated that in ARC-NPY neurons, decreasing extracellular glucose from 10 to 1 mM significantly increases AMP-activated kinase α2 (AMPKα2) phosphorylation within 2 min, acutely activating it. Concomitantly, lowered [glucose] or exposure to an AMPKα2 agonist increases phosphorylation of GSK3β, inhibiting its activity. Chen et al. (2012) also found lowered extracellular [glucose] stimulates an approximate 25% rise in [Ca^2+^]*_i_* and increases P/Q current selectively by ∼40%, while inhibition of AMPKα2 decreases VGCC currents of dissociated ARC-NPY neurons [160]. Consistent with GSK3β phosphorylation by AMPKα2 mediating rises in [Ca^2+^]*_i_*, exposure to LiCl_2_ during a drop in [glucose] enhanced increases in [Ca^2+^]*i*. AMPKα2’s actions are physiologically relevant since the ARC-NPY neurons play a central role in regulating food intake and glucose homeostasis [161,162]. Moreover, recent kinase pathway analysis of iPSC-derived glutamatergic neurons from a patient with a 4-bp mutation in DISC1 found that DISC1 mutant cells had significantly lower AMPKα2 levels compared with neurons derived from a wildtype sibling. These findings reveal a possible role for DISC1 in regulating AMPKα2 [11]. Whether DISC1 also plays a role in regulating P/Q currents in ARC-NPY nucleus neurons has not been examined. Nevertheless, the findings in ARC-NPY and hippocampal neurons suggest a convergent common pathway of action where GSK3β inhibition by AMPKα2 and/or DISC1 relieves P/Q-VGCCs from tonic inhibition.

While Zhu et al. (2010) found active GSK3β decreases P/Q current, SNARE protein association with channels and with each other, as well as SV fusion, they did not probe whether DISC1 plays a role in regulating exocytosis [148]. However, using RNAi technology and a DISC1 knockout mouse model, Tang et al. (2016) interrogated DISC1’s role in activity-dependent neurotransmitter release [78]. SV fusion was imaged by electrophoresing dissociated hippocampal neurons (14–16 DIV) with the synaptic tracer vGpH, a mutant pH-sensitive GFP (pHluorin) [163], fused to the SV glutamate transporter, vGlut1 [78]. At rest, pHluorin faces the acidic lumen of SVs. However, after SV fusion, vGpH undergoes a ∼20-fold increase in fluorescence intensity in response to the neutral pH of the extracellular solution [164]. Following glutamate exocytosis and vGpH re-uptake, SVs rapidly re-acidify and consequently vGpH fluorescence is quenched. The pH-sensitive properties of vGpH make it a valuable tool for monitoring SV lifecycle at single synapses.

By measuring changes in fluorescence during field stimulation, Tang et al. (2016) imaged hundreds of individual synaptic boutons from cultured rat hippocampal neurons transfected with shRNA targeting *Disc1’s* exon 2 or 9 [78]. With either shRNA construct, neurons exhibited a slower rise time and lower intensity of vGpH signal during both an initial and second 10 Hz/300 action potential (AP) stimulation period relative to neurons expressing an scr-shRNA. If stimulation duration was increased to 1200 APs, the vGpH response reached the same maximal level for scr- and shRNA-treated neurons, suggesting DISC1 knockdown did not affect the total releasable pool of SVs or the rate of membrane recovery (a measure of endocytosis), but did slow the kinetics of SV fusion. Cultured hippocampal neurons from a mouse model with exons 2 and 3 deleted [114] lack full-length DISC1, the major isoform in mouse brain. These neurons show similar SV cycling defects where the rates and amplitudes of vGpH signals were reduced compared to wildtype neurons. Together these findings indicate that loss of full length DISC1 disrupts rapid exocytosis of SVs from glutamatergic neurons with no obvious effect on the total releasable pool. For these experiments, Tang et al. (2016) examined whether AP-evoked intracellular Ca^2+^ signals changed with decreased DISC1 [78]. The authors used the SV-targeted Ca^2+^ sensor SyGCamp3 [165] to detect changes in Ca^2+^ levels during a 10 Hz train of 300 APs and found a blunted Ca^2+^ signal in the absence of full length DISC1 expression. Elevating extracellular Ca^2+^ from 2 to 4 mM Ca^2+^ increased AP induced Ca^2+^ signals and restored the vGpH response suggesting a possible action of DISC1 on VGCCs.

In central synapses Ca_V_2.1 (P/Q-VGCCs) and Ca_V_2.2 (N-VGCCs) largely control excitation–secretion coupling [145,158]. Tang et al (2016) determined that with their AP stimulation protocol, blocking Ca_V_2.2 with ω-conotoxin decreased vGpH signals ∼73% while inhibiting Ca_V_2.1 with ω-agatoxin TK decreased the signal by 42% in cultured hippocampal neurons [78]. Perhaps not surprisingly, blocking Ca_V_2.2 occluded the previously observed decrease in exocytosis, when comparing control to DISC1 knockdown neurons. These findings suggest that under these stimulation conditions, DISC1 regulates both Ca_V_2.1 and Ca_V_2.2 mediated SV release from hippocampal neurons with Ca_V_2.2 activity largely responsible for excitation–secretion coupling. Surprisingly, no difference in the fraction of boutons containing Ca_V_2.2 or in the intensity of Ca_V_2.2 labeling in presynaptic boutons were found. The same held true for Ca_V_2.1. Despite the super resolution images, the detailed images appear insufficient to rule out the possibility that DISC1 regulates the number of functional Ca_V_2 channels expressed in membrane associated with active zones.

Therefore, to test for this possibility, Tang et al. (2016) examined the effect of DISC1 on Ca_V_2.2 currents using whole-cell patch clamp methods [78]. They first cotransfected DISC1 with Ca_V_2.2 and axillary subunits into HEK293 cells and found that the presence of DISC1 significantly increased both peak and tail current density with no change in voltage-dependence of activation nor any obvious change in opening kinetics. Recordings of Ca_V_2.1 in cells expressing DISC1 exhibited a similar voltage-independent potentiation of the whole-cell current. These findings are consistent with DISC1 promoting cell surface expression of functional Ca_V_2.1/2.2 VGCCs at least in HEK293 cells with no change in channel gating. It would be useful to determine whether similar changes in whole-cell currents occur in hippocampal neurons as well since the fluorescent images appear to show no change in the percent of synaptic boutons expressing Ca_V_2 VGCCs. Despite the number of Ca_V_2-positive boutons remaining unchanged as well as the intensity of Ca_V_2.2 staining in nerve terminals, their whole-cell recordings from HEK293 cells suggest a significant decrease in functional channels involved in transmitter exocytosis. These seemingly conflicting findings may result simply from insufficient resolution for observing a change in staining intensity in the plasmalemma of the hippocampal boutons. Resolving the apparent difference in findings will provide a better understanding of the mechanism by which DISC1 increases VGCC activity and thus neurotransmitter release. Despite this shortcoming, Tang et al. (2016) is the first report to document VGCC regulation by DISC1 in neurons [78]. Moreover, their findings show that the actions of DISC1 overexpression on VGCCs parallel those of decreasing GSK3β activity in hippocampal neurons [148,155], and ARC neurons [160]. Whether they act in concert within an active zone signaling microdomain awaits further interrogation.

## Concluding remarks and remaining unanswered questions for understanding the cellular basis of mental illness using the DISC1 model

Since the initial description of a Scottish family with high penetrance for MMI and subsequent identification of a translocation defect *t*(1:11) in affected individuals, a tremendous amount of information is now known about DISC1’s actions at the cellular level and its requirement for normal cognitive functioning. DISC1’s influence on psychiatric illness appears quite broad since it interacts with so many proteins. This review has focused on DISC1’s interactions with proteins that affect Ca^2+^ signaling. The need to fully understand DISC1’s function on cognition will continue to drive research to confirm DISC1 protein expression, understand the functional importance of these different DISC1 splice variants, identify DISC1’s many binding partners, and then interrogate them to better understand DISC1’s regulatory actions on each protein in different types of neurons over time. A common functional theme in adult neurons is that DISC1 regulates movement at the molecular level. Specifically, DISC1 acts as a gate keeper for the movement of a variety of molecules and organelles: mRNA, RNA particles, SV, and mitochondria, by tethering molecules and organelles to molecular motors. Additionally, by dynamically regulating Ca^2+^ flux through IP_3_R1 at MAMs, critical for proper mitochondrial functioning, DISC1 may control local energy production required for neurite branching, elongation, and synaptogenesis. This realization suggests that one pathology with *tr*DISC1 may be due to excessive Ca^2+^ influx into mitochondria, compromising coupling between its electrical potential and ATP production. DISC1 regulates additional Ca^2+^ physiology by controlling the movement of VGCC subunit mRNA into dendrites and axons, which is predicted to affect their location and expression levels over time and consequently alter excitation-translation coupling occurring at synapses. At adult synapses, DISC1 increases, while GSK3β decreases, VGCC expression to reciprocally tune neurotransmitter release. Whether DISC1, GSK3β, AMPKα, and other known binding partners exist together in a microdomain with VGCCs at or near SV fusion sites is a critical question that remains to be answered.

Indeed, many questions remain about the exact relationship between DISC1 and VGCCs. We are especially interested in knowing whether DISC1 directly interacts with particular VGCC subunits at synapses. Additionally, careful characterization of where and when different splice variants of DISC1 are expressed should allow the field to answer the question of whether loss of one function, or all of them, contribute to MMI susceptibility with expression of *tr*DISC1. Moreover, it is possible that altered splice variant expression ratios in certain cell types during development and into adulthood increase susceptibility to MMI, independently of DISC1 truncation. This could arise from disruption in the normal processes mediating gene splicing rather than a change in DISC1 sequence. Similarly, various combinations of SNPs in DISC1 may be required to elevate MMI susceptibility [85]. These broad questions will take a great deal of interrogation before arriving at answers. However, the increase in DISC1 mouse models, commercially available antibodies, and a host of new nanotechnologies for studying molecules in single central neurons should allow the field to probe further how *tr*DISC1 alters Ca^2+^ signaling via Ca^2+^ channels resulting in increased susceptibility for developing MMI. Current cell and molecular studies, summarized in this review, highlight a new appreciation of a DISC1-Ca^2+^ signaling node as critical for adult cognition.

## Data Availability

There are no primary data included in the manuscript as it is a review.

## Abbreviations

AMPKα2: AMP-activated kinase α2
AP: action potential
ARC-NPY: NPY-expressing arcuate nucleus
ARM: arginine-rich motif
BPD: bipolar disorder
CNV: copy number variation
DISC1: Disrupted in Schizophrenia 1
ER: endoplasmic reticulum
ERP: event-related potential
GABA: gamma-aminobutyric acid
GSK3β: glycogen synthase kinase 3β
GWAS: genome-wide association study
HZF: hematopoietic zinc finger protein
IP_3_R: inositol 1,4,5 triphosphate receptor
iPSC: induced pluripotent stem cell
MAM: mitochondria-associated ER membrane
MDD: major depression disorder
MMI: major mental illness
NPY: neuropeptide Y
ROS: reactive oxygen species
Schz: schizophrenia
SNP: single nucleotide polymorphism
SNPH: syntaphilin
SV: synaptic vesicle
VGCC: voltage-gated Ca^2+^ channel
